# Fine mapping and identification of the gene *Cla019481* responsible for patches at the hilum on the testa of watermelon seeds

**DOI:** 10.3389/fpls.2025.1680623

**Published:** 2025-12-09

**Authors:** Runbu Lv, Dan Zhou, Hongyuan Liu, Chengyuan Xie, Yufu Zhang, Xiaoyu Zhang, Dongye Li, Shuangwu Ma, Jifeng Chen

**Affiliations:** 1 School of Life Sciences, Zhengzhou University, Zhengzhou, Henan, China; 2 Zhengzhou Fruit Research Institute, Chinese Academy of Agricultural Sciences, Zhengzhou, Henan, China; 3 National Supercomputing Center in Zhengzhou, Zhengzhou University, Zhengzhou, Henan, China

**Keywords:** *Citrullus lanatus*, *Cla019481*, patches at hilum, INDEL, QTL, SNP, qRT-PCR

## Abstract

The presence or absence of patches at the seed hilum is a valuable phenotypic marker for breeding new cultivars and identifying watermelon germplasm resources, although the candidate gene regulating this trait remains unknown. In this study, the F_1_ generation seeds (with patches at the hilum) were derived from a cross between the female parent (no patches at the hilum) and the male parent (with patches at the hilum), and a back cross (BC) population was generated by crossing F_1_ with the female parent. The segregation ratio of the patches and no-patches trait conforms to the expected 1:1 Mendelian ratio in the BC population. Restriction-site–associated DNA sequencing was performed on the BC population to construct a high-density genetic map. The analysis revealed a major quantitative trait locus (QTL) on chromosome 3, spanning 5,375,019–5,784,364 bp and harboring 35 annotated genes from *Cla019451* to *Cla019485*, which govern the stably inherited trait of patches at the hilum on the testa of watermelon seeds. A reliable derived cleaved amplified polymorphic sequence (dCAPS) marker was developed within the interval, demonstrating perfect genotype–phenotype co-segregation. Consequently, the target QTL was delimited to a 40-kb region on chromosome 3, which contains the candidate gene *Cla019481* for patches at the hilum. Insertions/deletions (Indel) and single-nucleotide polymorphisms (SNPs) indicated that *Cla019481* was the top candidate gene responsible for the presence or absence of patches at the hilum. Based on dCAPS marker development for SNP genotype identification and visual phenotype classification across different groups of watermelon accessions, no phenotypic inconsistencies were observe in materials lacking patches at the hilum. In other words, the genotype indicated absence of patches at the hilum, and the phenotype corresponded accordingly in the tested materials. Gene expression validation experiments using materials with/without patches at the hilum, combined with quantitative RT-PCR (qRT-PCR), revealed a positive correlation. Elevated *Cla019481* expression coincided with progressive darkening of hilum pigmentation during the three seed development stages (8, 18, and 25 days after flowering). The verification test results demonstrate that *Cla019481* expression critically regulates hilum formation. *Cla019481* thus plays a significant role in the presence of patches at the hilum on watermelon seeds.

## Highlights

We revealed that the gene *Cla019481* is located within a major QTL on chromosome 3, controlling the presence of patches at the hilum on the testa of watermelon seeds.

## 1 Introduction

Watermelon (*Citrullus lanatus*) is a globally significant cucurbit crop, cultivated extensively in warm regions, particularly in China and other fruit-producing countries (Rahimi and Abdolinasab, 2022). The fleshy fruit provides essential nutrients (Guo et al., 2013) and contains citrulline (Akashi et al., 2001; Wu et al., 2007), a compound with documented antioxidant and vasodilatory functions in humans (Rimando and Perkins-Veazie, 2005). Numerous studies have investigated plant traits, especially seed coat-related traits in watermelon (Paudel et al., 2019; Guo et al., 2021) and other crops, including soybean seed coat color (Yang et al., 2023), testa and hilum color in pea (Konstantopoulos et al., 2023) and soybean (Kaňovská et al., 2024), soybean hilum color (Zhou et al., 2024), and seed coat color in *Brassica napus* (Li et al., 2024), sesame (Elsafy et al., 2025), common bean (*Phaseolus vulgaris* L.) (Plestenjak et al., 2025; Roy et al., 2025), wheat (Afonnikova et al., 2024), and peanut (Zhao et al., 2020). The spotted, dark-hilumed phenotype is an important trait for breeding/selecting new cultivars for fresh-eating watermelon or for seed production for edible seed consumption. To date, no research on this trait has been published.

In plant trait research, restriction-site–associated DNA sequencing and various other molecular biological techniques have been applied. Many genetic traits have been investigated using quantitative trait locus (QTL) mapping in watermelon and other crops. Li et al. revealed a major QTL controlling watermelon seed size in an F_2_ population and successfully narrowed down the physical interval to four genes (Li et al., 2018b). QTL mapping has also been conducted for ovary traits (Amanullah et al., 2020) and for pericarp and fruit-related traits in melon (*Cucumis melo*) (Zhang et al., 2020). Using H-7 and SA-1 as experimental materials, QTLs for rind hardness were mapped to linkage group 4, and red flesh color was mapped to groups of 2 and 8 (Hashizume et al., 2003). Othe studies have identified QTLs for watermelon rind color (Li et al., 2019), flesh color (Gusmini and Wehner, 2006; Bang et al., 2007), lycopene content (Liu et al., 2014), sugar accumulation (Ren et al., 2014, Ren et al., 2018), bitterness (Shang et al., 2020), and overall fruit characteristics (Sandlin et al., 2012). Complementary studies in cucumber (*Cucumis sativus*) revealed QTLs controlling flowering time and fruit dimensions (Sheng et al., 2020).

For watermelon seed coat color, the genes at the *R*, *T*^1^*, W*, and *D* loci were mapped on chromosomes 3, 5, 6, and 8, respectively, using QTL-seq and genotyping-by-sequencing (Paudel et al., 2019). Through genome-wide association analysis, one QTL associated with seed coat color was detected on chromosome 10, and five QTLs associated with seed coat patches were detected on chromosomes 2, 6, 7, 10, and 11, respectively (Guo et al., 2021). For lobed leaf traits, the genes *ORF18* and *ORF22* were narrowed down to a physical distance of 127.6 kb and considered candidate genes in watermelon, as confirmed by quantitative RT-PCR (qRT-PCR) (Wei et al., 2017). Using field data from an F_2:3_ segregating population and molecular markers for the F_2_ population, a total of 10 QTLs were identified for watermelon fruit quality traits (Cheng et al., 2016). The major QTL for *Fusarium oxysporum* f. sp. niveum (FON) race 1 resistance was found to be on chromosome 1, and FON race 2 resistance on chromosomes 9 and 10 (Ren et al., 2015). Lambel et al. (2014) identified the QTL for resistance to *Fusarium oxysporum* race 1 via genotyping-by-sequencing and fine-mapped an interval of 2.3 to 8.4 cM on chromosome 1. QTLs have also been applied to study powdery mildew resistance, seed size, and fruit shape (Kim et al., 2015). Collectively, these studies demonstrate that QTL mapping is a robust methodology for dissecting agronomic traits.

Recent advances in molecular markers and sequencing technologies have enabled the precise identification of genetic loci and candidate genes underlying agronomic traits in plants. Next-generation sequencing facilitates the construction of high-density genetic maps (Shi et al., 2014; Shang et al., 2016; Wang et al., 2017b; Feng et al., 2023) and genome-wide gene analysis in crops (Feng et al., 2023). In watermelon, these approaches have successfully mapped fruit traits (Li et al., 2018a), including yellow skin pigmentation (Dou et al., 2018a), fruit shape (Dou et al., 2018b), and other traits. Next-generation sequencing-based genotyping also enables the identification of single-nucleotide polymorphisms (SNPs), which are used to construct genetic linkage map. SNP markers are widely applicable for trait dissection, as demonstrated in studies of fruit yield components (Katuuramu et al., 2023a), soluble solids content, flesh color, and fruit shape in citron watermelon (Katuuramu et al., 2023b), and watermelon seed coat color (Paudel et al., 2019).

Previous studies have indicated that gene expression levels influence seed coat color and are related to polyphenol oxidase (PPO), which is often associated with browning (Kampatsikas et al., 2017; Taranto et al., 2017) and petal color formation (Nakayamaa et al., 2001; Wang et al., 2024). The expression level of the gene *MC03g0810* shows a positive correlation with PPO activity in the black testa of bitter gourd (*Momordica* spp.) (Zhong et al., 2022), and the gene *Cla019481* has been identified as a candidate gene for melanin accumulation in the black seed coat of watermelon (Li et al., 2020). Therefore, the dark color of patches at the watermelon seed hilum is likely related to gene expression during seed development stages.

**Figure 1:**
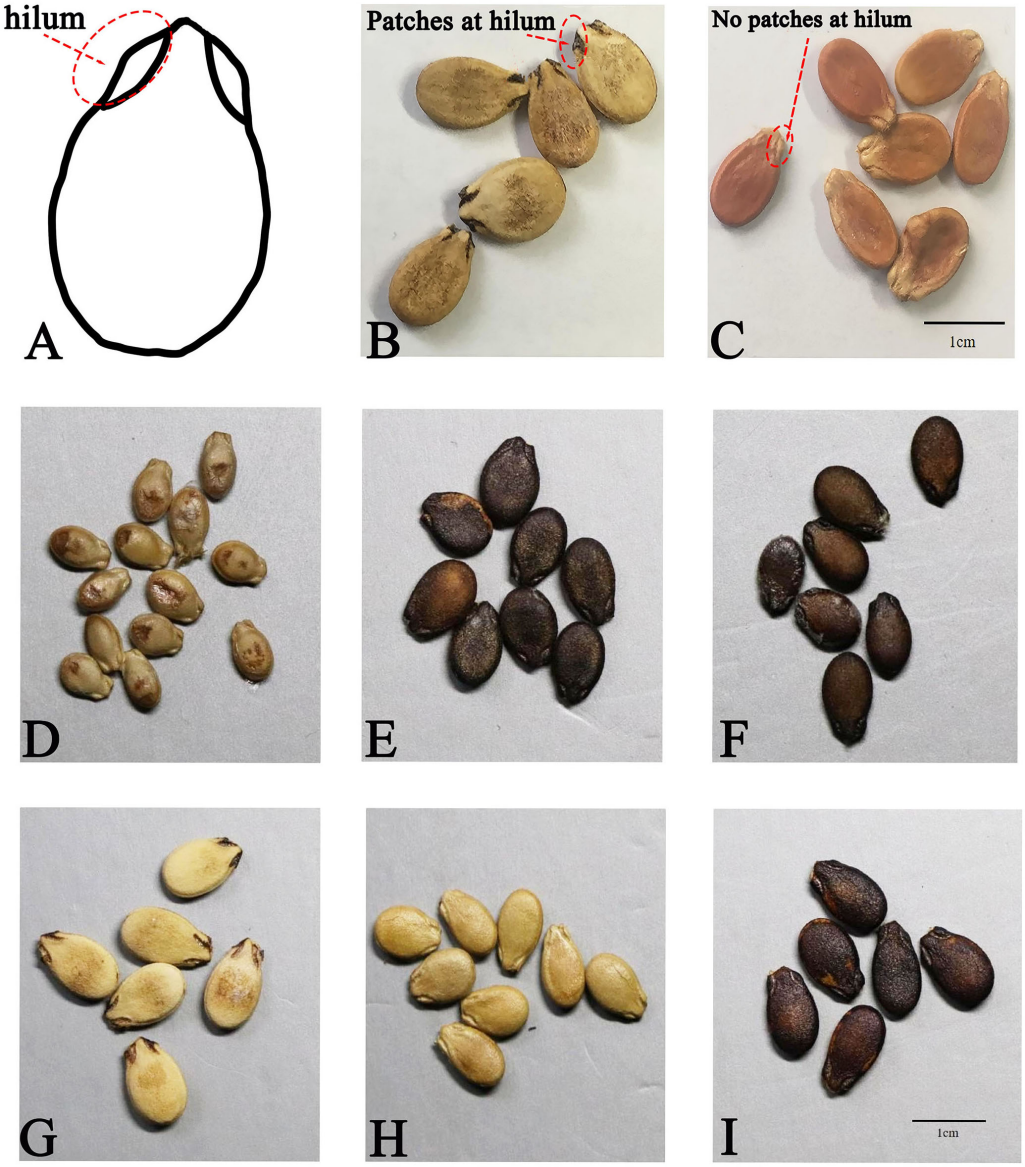
Phenotype information of patches at the hilum on the testa of watermelon seeds. **(A)** Location of the hilum on a watermelon seed diagram. **(B)** Seeds with patches at the hilum on the testa. **(C)** Seeds without patches at the hilum on the testa. **(D)** Female parent, B132, without patches at the hilum on the testa. **(E)** Male parent, B135, with patches at the hilum on the testa. **(F)** F_1_ generation, B139, with patches at the hilum on the testa. **(G)** 17CB243 of the BC population, with patches at the hilum on the testa. **(H)** 17CB222 of the BC population, without patches at the hilum on the testa. **(I)** 17CB276 of the BC population, seeds for which patches at the hilum on the testa could not be determined.

Seeds play a crucial role in the watermelon life cycle. Through domestication and cultivation, watermelon has undergone extensive natural and artificial selection, resulting in diverse varietal characteristics. According to Ma (2005), the “hilum” is defined as the eye-like pattern on both sides of the rostral part of watermelon seed. The dark color at the hilum, when visually inconsistent with the color of the seed surface, is referred to as “patches at the hilum.” **Figure 1A** shows the hilum position on a schematic diagram of a watermelon seed, **Figure 1B** shows the position of the patch-hilum, and **Figure 1C** shows seeds with and without patches at the hilum in photos of watermelon seeds. Systematic evaluation of germplasm at the National Mid-term GenBank for Watermelon and Melon (Zhengzhou, China) revealed that the patch-hilum trait is ubiquitous in cultivated watermelon varieties but rare in wild types (data unpublished). Therefore, we propose that the presence/absence of patches at the hilum serves as a robust phenotypic marker for authenticating watermelon genetic resources and tracking domestication signatures in germplasm collections. Despite its diagnostic value, no genetic markers or candidate genes underlying this trait have been identified to date.

## 2 Materials and methods

### 2.1 Plant materials

The F_1_ generation seeds of B139 were derived from a cross between B132 (the female parent, no patches at the hilum) and B135 (the male parent, with patches at the hilum). A backcross (BC) population of 98 individuals (**Supplementary File ST1-B1**) was generated by crossing F_1_ with the female parent B132. Seventy-one watermelon accessions were used as representatives of local varieties, breeding cultivars/lines, and wild-type germplasm of watermelon (**Supplementary File ST1-B2**). Four watermelon materials used for qRT-PCR were 17Q140-18Z and 17QB47, both with no patches at the hilum, and 18CB83 and 18CB90, both with patches at the hilum (**Supplementary File ST1-B3**). All of these watermelon materials were from germplasm resources in the National Mid-term GenBank for Watermelon and Melon (Zhengzhou, China) (https://www.cgris.net/home).

### 2.2 Analysis of the patch-hilum trait and DNA extraction

Mature watermelon fruits were harvested 35–40 days postpollination, and seeds of all populations were collected for visual phenotypic classification, based on the presence or absence of patches at the hilum. Phenotypic screening was performed through manual visual observation by personnel with expertise in watermelon germplasm resources and breeding practices. Young leaves from parental plants, F_1_ and BC population (**Supplementary File ST1-B1**), were sampled for QTL mapping and genotyping. Segregation ratios were evaluated using the Chi-square test (*χ*^2^ test) in SPSS Statistics 25.0 (IBM Corp., Armonk, NY, USA). Genomic DNA was extracted from fresh leaf tissues via a modified CTAB method (Li et al., 2009). DNA was quantified with a NanDrop-1000 spectrophotometer (NanoDrop, Wilmington, DE, USA) and evaluated by electrophoresis in a 1.0% agarose gel.

### 2.3 Map construction and QTL analysis

The parent plants and 98 BC population were sequenced using 150-nucleotide paired-end sequencing on the Illumina HiSeq™ platform, and the reads were aligned to the watermelon reference genome (97103) v2 (http://www.icugi.org/organism/21). For constructing the genetic map using SNP markers (Murray and Thompson, 1980), MSTmap software was applied. After filtering out low-quality data to obtain clean reads (*Q* ≥ 30), the sequences were compared with published references using the BWA software (Li and Durbin, 2009). GATK software (the Genome Analysis Toolkit, Broad Institute, Cambridge, MA, USA) was applied for SNP detection, and the vcfutils tool of SAMtools (Wang et al., 2017a) was used for identifying high-quality mutation sites. To determine different molecular markers, all SNPs and insertion/deletion (Indel) markers were detected by parental polymorphism markers. Marker encoding was based on parental polymorphism (e.g., AA and CC parents; GT/TG progeny was classified as missing data). Three criteria were used to filter out invalid SNPs: (1) exclusion of sites absent in parental genotypes, (2) removal of loci with > 50% missing data across progeny, and (3) elimination of markers showing segregation distortion (*p* < 0.05). The retained high-confidence SNPs were used for final map construction.

For grouping the linkage, a single-link clustering method was used, referencing the genomic information. First, the controlling logarithm of odds (LOD) threshold, ranging from 4.0 to 20.0, was applied to ensure that the number of linkage groups corresponded to the species’ chromosomal count. Second, scaffold integrity and chromosomal collinearity were preserved across all linkage groups (LGs), such that all SNPs derived from a single genomic scaffold co-localized to one LG, and genetic map clustering exhibited minimal conflict with the reference genome assembly.

We performed QTL analysis via composite interval mapping implemented in the R/qtl package (Broman et al., 2003). LOD thresholds for significant QTL were established by the permutation test (parameter = 1,000). QTL intervals were defined with a minimum 80% support interval. The location of the peak LOD value was designated as the major QTL locus within the significant interval. The linkage map was generated by using the Kosambi mapping function, which converted the recombination frequencies into map distances in centimorgans (cM). MapGene2Chromosome v2 software (http://mg2c.iask.in/mg2c_v2.0/) was used for chromosome (Chr) and LG maps, and Microsoft Paint was used for the other schematic diagrams.

### 2.4 Marker development and genotype identification

A total of 96 individuals (**Supplementary File ST1-B1**) underwent genotyping analysis, comprising 94 BC progeny (excluding four individuals with visually undetermined seeds, black testa; **Figure 1I**) and two validation accessions, B5–8 and 18CB83 (both exhibiting patches at the hilum visually).

Based on watermelon genome data (http://www.icugi.org/), about 500 bp of the Indel locus and selected SNP locus were extracted using the Perl language script compiled by the laboratory. The CAPS/derived cleaved amplified polymorphic sequence (dCAPS) primers were designed using the online analysis software dCAPS Finder 2.0 (http://helix.wustl.edu/dcaps/dcaps.html) and Oligo 7 software. The commonly used restriction endonucleases (e.g., *EcoR*I, *Taq*I, *Alu*I, *Hind*III, *Rsa*I, *Mse*I) were employed. Primer Premier 5 software was used for primer design, and the designed primer sequences were synthesized by Bioengineering Engineering Co. Ltd., Shanghai, China.

While converting Indels and SNPs in the QTL region to PCR-based markers for fast and reliable analysis, the respective PCR primer sets (Indel and dCAPS) (**Supplementary File ST2**) were developed for genotyping and polymorphism screening across 96 individuals (**Supplementary File ST1-B1**). PCR amplification was performed in a 25-μl reaction containing 1 μl of 200 ng genomic DNA, 12.5 μl of 2× Power Taq PCR Master Mix, 1 μl of 10 µM per primer (forward/reverse), and 9.5 μl of RNase-free water. The thermal cycling conditions were as follows: predenaturation at 94°C for 5 min; 35 cycles of 94°C for 20 s, 55°C for 1 min, and 72°C for 30 s; and a final extension at 72°C for 5 min, followed by a hold at 4°C. For CAPS/dCAPS digestion, 5 μl of PCR product was incubated with 1.5 μl of 10× reaction buffer, 0.5 μl of restriction endonuclease (Thermo Scientific™, Waltham, MA, USA), and 8 μl of RNase-free water (15 μl total volume) at 37°C for 12 h. The digestion products were resolved by 8% polyacrylamide gel electrophoresis at 260 V for 40 min, silver-stained for 15 min, rinsed, fixed in sodium thiosulfate for 2 min, and visualized using X-ray film imaging (Bio-Rad^®^ Gel Doc™ XR+, Hercules, CA, USA). The markers with polymorphism were used for fine mapping.

### 2.5 Candidate gene prediction and gene expression analysis by qRT-PCR

The QTL interval was gradually narrowed down within the final mapping range of the candidate gene using the verification of molecular markers, and the coding sequences were found referencing the watermelon genome database (http://www.icugi.org/). The candidate gene functions were retrieved from NCBI using the BLASTp tool (https://blast.ncbi.nlm.nih.gov/Blast.cgi). The specific primers of the candidate genes were designed using Primer Premier 5, and the housekeeping actin gene (**Supplementary File ST3**) was used as an internal control (Kong et al., 2014) for qRT-PCR based on the Cucurbit Genomic Database (http://cucurbitgenomics.org).

For gene expression, the watermelon seeds were collected at three growth stages, which were 8 days after flowering (DAF), 18DAF, and 25DAF. RNA was extracted from the collected seeds using the RNAprep Pure Polysaccharide Plant Total RNA Extraction Kit (TIANGEN, Beijing, China), and RNA was reverse transcribed (RT) into complementary DNA (cDNA) using the Transcriptor First Strand cDNA Synthesis Kit (Roche, Basel, Switzerland). Each cDNA product (3 μl containing 200 ng) was diluted 15-fold by adding 42 μl of nuclease-free water. The qRT-PCR reaction (10 μl total volume) contained 1 μl of diluted cDNA, 0.25 μl of 10 μM each primer, 5 μl of SYBR Green I Master Mix 5, and 3.5 μl of RNase-free water. The mixture was set to the following conditions to obtain CT values for every sample: 95°C for 5 min (initial activation), 45 cycles of 95°C for 30 s, 55°C for 30 s, and 72°C for 35 s (amplification); followed by melt curve analysis at 95°C for 1 min, 60°C for 1 min, and 95°C for 10 min.

The relative expression level of the candidate gene *Cla019481* was determined using qRT-PCR with specific primers (**Supplementary File ST3**). Three independent biological replicates were prepared for each sample. The expression of the target gene was estimated by qRT-PCR (Nawaz et al., 2018) using the LightCycler^®^ 480 system (Roche, Switzerland), and the CT values from reactions in triplicate for each sample were calculated with the 2^−ΔΔCt^ method (Livak and Schmittgen, 2001). The significant difference analysis was performed using SPSS 25 software.

## 3 Results and discussion

### 3.1 Morphological analysis of patches at the hilum in the BC population

The testa color of B132 (female parent) was yellowish-brown without patches at the hilum (**Figure 1D**), and the testa color of B135 (male parent) was dark with patches at the hilum (**Figure 1E**). The F_1_ generation of B139 displayed uniformly dark testa with hilum patches (**Figure 1F**). Among the 98 BC population (**Supplementary File ST1-B1**), there were 46 accessions that produced seeds with patches at the hilum (**Figure 1G**), 48 accessions that produced seeds without patches at the hilum (**Figure 1H**), and four accessions that were unable to be determined because of the dark testa and dark hilum (**Figure 1I**). The segregation ratio (with patches: without patches = 46:48) conformed to the expected 1:1 Mendelian ratio (*χ*^2^ = 0.04, *p* = 0.84) (**Table 1**) (Zhang, 2019). The results indicated that a single autosomal dominant gene with a major-effect QTL in the BC population controlled patches at the hilum of watermelon seeds. The 46 patch-hilum accessions and 48 without-patch-at-the-hilum individuals of the BC population (the 94 accessions of the BC population) will be used in genotype–phenotype analysis later.

**Table 1:** Information on patches at the hilum of watermelon seeds in different groups.

Group	Plant No.	Patches at the hilum	No patches at the hilum	Unable to determine (black testa)	Expectation ratio	Chi-square value (*χ*^2^)	*p*-value
P_1_(B132)	40	0	40	–	–	–	–
P_2_ (B135)	41	41	0	–	–	–	–
F_1_ (B139)	22	22	0	–	–	–	–
BC	98	46	48	4	1:1	0.04	0.84

P_1_, female parent (B132) without patches at the hilum; P_2_, male parent (B135) with patches at the hilum; F_1_, hybrid generation (B139) with patches at the hilum; BC, back cross-population, 46 of 98 plants with patches at the hilum. The expected ratio is approximately 1:1.

### 3.2 QTL mapping of the patch-hilum trait

Whole-genome sequencing was performed on 100 plant accessions, including parental lines B132 and B135, and 98 BC progeny for genotyping. A total of 896.39 million reads were generated, averaging 10 million reads per individual, of which 94% showed high quality (*Q* ≥ 30). High-throughput sequencing at × 22 depth was conducted, resulting in the detection of 577,866 SNPs and 232,762 Indels between the parental lines. To refine the target QTL interval, reliable Indels and SNP loci were identified within the physical region of the QTL map. Bioinformatics analysis of high-quality reads (*Q* ≥ 30) yielded 515,688 SNP loci. After parental polymorphism screening and removal of markers with segregation distortion (*χ*^2^ test, *p* < 0.05), a final set of 7,662 SNPs was retained for linkage analysis.

**Figure 2:**
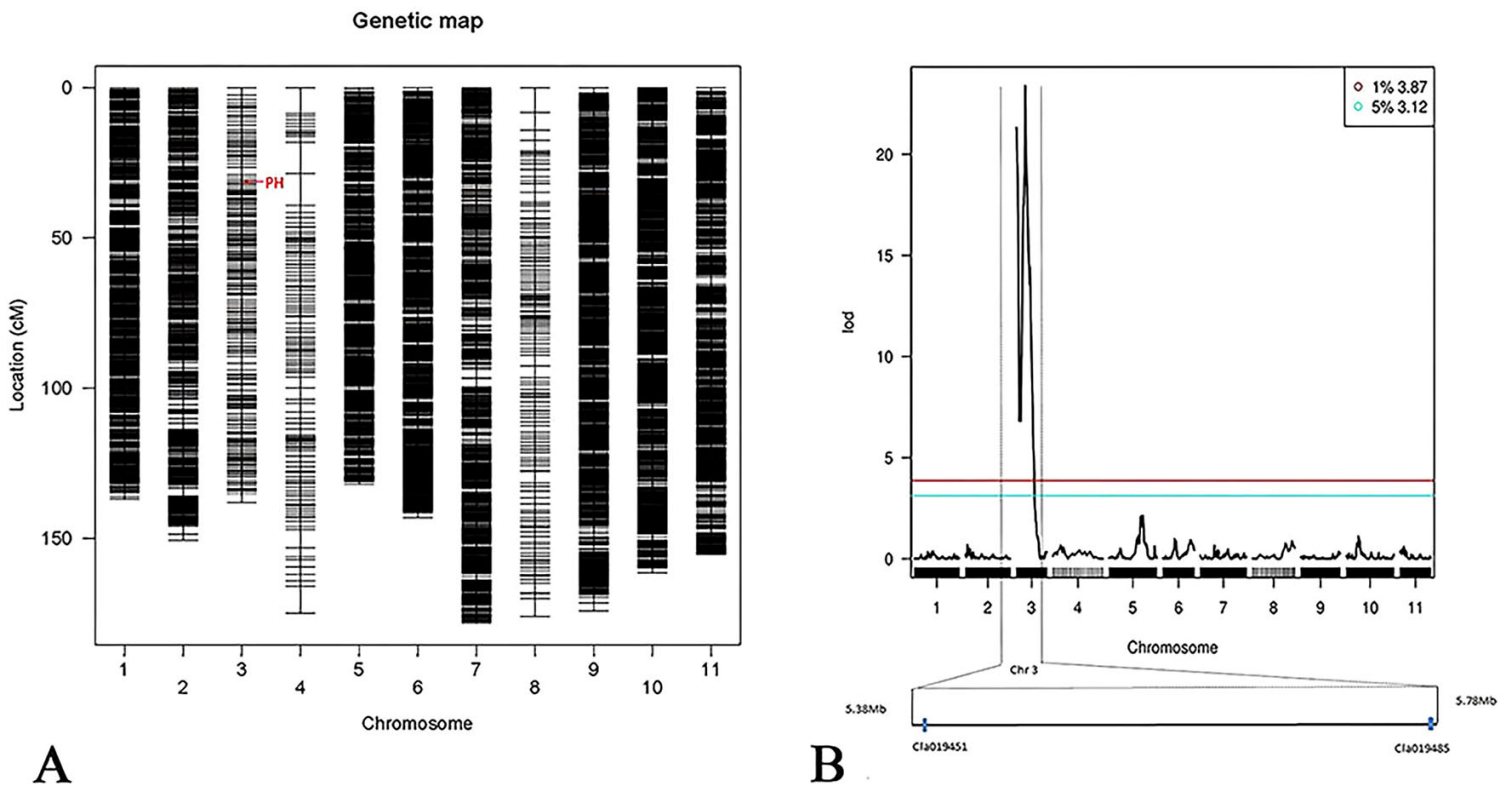
Genetic map for chromosome 3. **(A)** The resulting association for patches at the hilum is indicated on the watermelon chromosome 3, showing the region controlling the trait. The horizontal axis represents the watermelon chromosomes, with PH marking the gene location. **(B)** A locus for patches at the hilum of watermelon seeds was identified through QTL mapping. The PH gene is located on chromosome 3, within the region spanning *Cla019451~Cla019484* (5.38–5.78 Mb).

Using a single-linkage method and genomic information for linkage grouping, a high-density genetic map was constructed comprising 7,327 SNPs distributed across 11 linkage groups (**Figure 2A**). The total genetic distance was 1,811.91 cM, with 96% genome coverage and an average intermarker distance of 0.25 cM within each linkage group.

**Figure 3:**
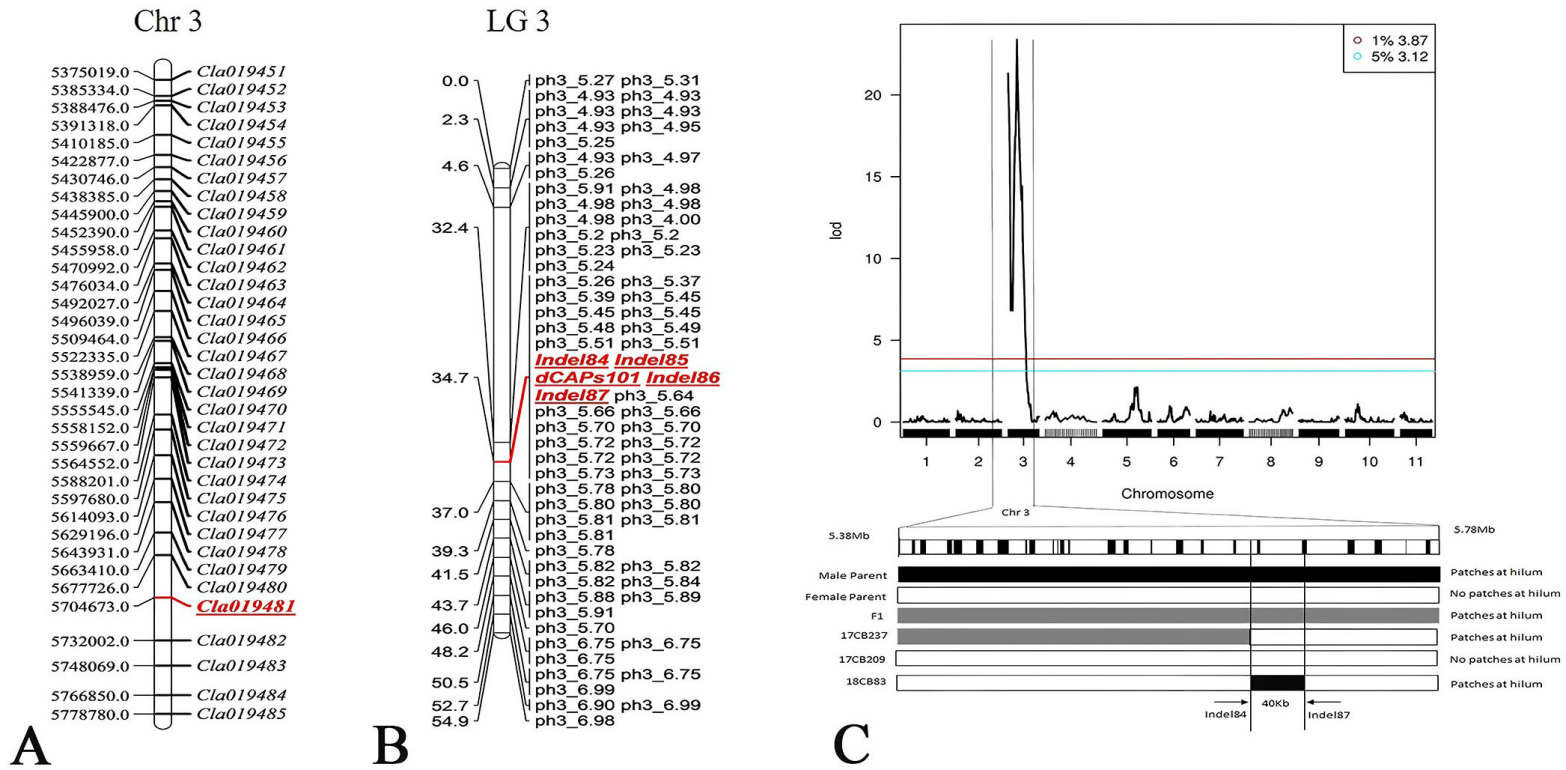
The gene loci and specific Indel and SNP markers at 34.7 cM within a 40-kb interval on chromosome 3. **(A)** The 35 gene loci in the region spanning 5,375,019–5,778,780 bp on watermelon chromosome 3, including gene *Cla019481*. **(B)** Consensus SNP and Indel markers for linkage group 3 (LG3) of *Citrullus lanatus*. Markers from Indel84 to Indel87, together with dCAPs101, cover an approximate genetic distance of 34.7 cM. **(C)** Two individuals (17CB237, 18CB83) of the 96 tested were estimated to carry the fine-mapped patches-at-the-hilum gene between markers Indel84 and Indel87, corresponding to a 40-kb interval on chromosome 3.

Combined with the high-density genetic linkage map and the phenotype data for the presence or absence of patches at the hilum in the BC population, the Rqtl-IM-binary method was applied to locate the QTL. A significant QTL was identified on chromosome 3, with a peak LOD score of 23.39, exceeding the 1% threshold of 3.87, and a confidence interval for the patch-hilum trait of 5,375,019–5,784,364 bp. This region harbors 35 annotated genes (*Cla019451* to *Cla019485*) (**Figures 2B**, **3A**). Using MapGene2Chromosome v2 (http://mg2c.iask.in/mg2c_v2.0/), the LG map and Chr map were visualized, highlighting the 5,375,019–5,784,364-bp region on chromosome 3 (Chr3) that contains *Cla019481* (**Figure 3A**), with a genetic distance of 54.9 cM on linkage group 3 (LG3) (**Figure 3B**). **Figure 3C** shows the LOD profile across the related region on Chr3 and the patch-hilum status pf different samples, indicating that the candidate gene controlling the patch-hilum trait in watermelon seed likely located within this interval.

### 3.3 Fine mapping the QTL region for the patch-hilum trait

To identify the locus controlling patches at the hilum in watermelon seeds, a population of 96 individuals was investigated, including 94 accessions in the BC population (excluding four undetermined individuals) and two other experimental materials (B5–8 and 18CB83, both with patches at the hilum) (**Supplementary File ST1-B1**). Genotypic analysis revealed that the accessions of 17CB237 and 18CB83 exhibited consistency between markers Indel84 and Indel87 (**Figure 3C**). A reliable dCAPS marker (dCAPS 101) was developed within this interval (**Figure 3B**), demonstrating perfect genotype–phenotype co-segregation. Consequently, the target QTL was delimited to a 40-kb region (physical position: 5.69–5.73 Mb) on Chr 3 (**Figure 3C**).

### 3.4 Identification of genes associated with the patch-hilum trait

Further analysis of the 40-kb genomic interval, based on the watermelon genome database (http://www.icugi.org/), identified one annotated gene, *Cla019481*, which was predicted to be a PPO gene containing InterPro domain IPR016213. Nucleotide sequence alignment revealed that *Cla019481* shares 78% identity with the *Trifolium pratense* PPO1/4 gene and 59% amino acid sequence identity with the *Momordica charantia* PPO protein. PPOs are ubiquitously expressed in plants (Mayer, 2006; Webb et al., 2014) and function in oxidation and color change. Since *Cla019481* is a homologous PPO gene in watermelon, it is likely responsible, at least in part, for the patches at the hilum on watermelon seeds.

**Figure 4:**
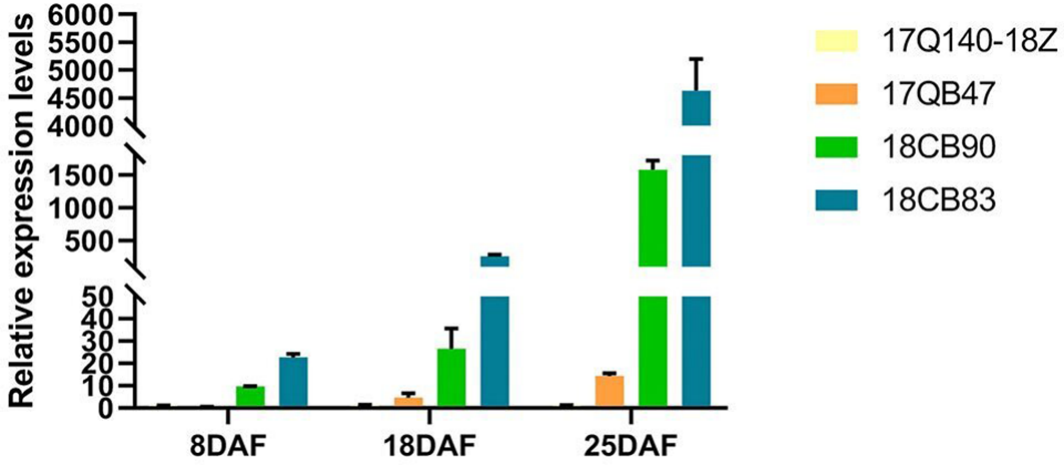
Relative expression levels of gene *Cla019481* during different seed developing periods in four materials. 17Q140-18Z and 17QB47 lack patches at the hilum, whereas 18CB90 and 18CB83 possess patches at the hilum. Error bars represent the mean ± standard deviations (SDs) of three biological replicates. DAF, days after flowering.

The expression pattern of the candidate gene *Cla019481* was validated across three seed developmental stages (8, 18, and 25DAF) in four watermelon accessions using qRT-PCR. These accessions included two nonpatch controls (17Q140-18Z and 17QB47) and two patch-hilum materials (18CB90 and 18CB83). *Cla019481* expression was significantly higher (*p* < 0.05) in patch-hilum materials at all developmental stages (**Figure 4**). These results confirm *Cla019481* as the candidate gene controlling the patch-hilum trait in watermelon seeds.

### 3.5 Genotype and phenotype consistency analysis in different groups of accessions

The dCAPS marker, named dCAPS101 (**Supplementary File ST2**), was designed based on the sequence information of *Cla019481*. To validate genotype–phenotype concordance, 96 individuals (**Supplementary File ST1-B1**), 71 watermelon accessions (**Supplementary File ST1-B2**), and the previous four individuals used for qRT-PCR (**Supplementary File ST1-B3**) were evaluated using dCAPS markers and human visual judgment for the patch-hilum trait. The results revealed 100% concordance between dCAPS101 genotypes and phenotypes in both the 96 individuals and the four individuals (**Table 2**; **Supplementary Files ST1-B1, B3**). Among the 71 accessions, 49 (69%) exhibited phenotypic consistency with dCAPS101-positive genotypes (**Table 3**; **Supplementary File ST1-B2**), while 22 with black testa and black hilum were marked “uncertain” in **Tables 2**, **3**. These 22 accessions are listed as “without patches at hilum” in **Supplementary File ST1-B2**. These results confirm that *Cla019481* functions as a key regulator of patch formation at the hilum in watermelon seeds.

**Table 2:** Consistency rate between genotype and phenotype in different material groups.

Material group	Patch-hilum info.	Genotype materials No.	Phenotype uncertain (black testa) No.	Consistent No.	Consistency rate (%)	Total consistency rate (%)
96 individuals	No	48	0	48	100	100
	Yes	48	0	48	100	
4 individuals	No	2	0	2	100	100
	Yes	2	0	2	100	
71 accessions	No	16	0	16	100	69
	Yes	55	22	33	60	

The genotype–phenotype consistency rate was 100% for 96 individuals and four individuals, and 69% for 71 accessions. Among the 55 genotype patch-hilum materials, 33 displayed a clear trait of patches at the hilum, while 22 had black testa and black hilum seeds, which were marked as uncertain.

**Table 3:** Genotype–phenotype consistency rate of the 71 accessions.

Genotype same as	Patches info.	Genotype material No.	Phenotype uncertain (black testa) No.	Consistency No.	Consistency rate (%)
A (B132, *P*_1_)	No	16	0	16	100
B (B135, *P*_2_)/H (B139, F_1_)	Yes	55	22	33	60
Total	–	71	22	49	69

A, genotype same as the female parent (B132, P_1_) without patches at the hilum; B, genotype same as the male parent (B135, P_2_), H, genotype same as F_1_ (B139) with patches at the hilum.

### 3.6 Patch-hilum trait of watermelon seeds

The black color of the testa occasionally obscured visual identification of patches at the hilum, particularly in accessions with dark testa and dark-hilum seeds (**Figures 1E, F, I**). F_1_ (B139) (**Figure 1F**), crossed back with female parent B132 (**Figure 1D**), produced a BC population exhibiting separated phenotypes of seeds with patches at the hilum (**Figure 1G**) and seeds without patches at the hilum (**Figure 1H**). For this reason, we considered the male parent (B135) (**Figure 1E**) and F_1_ (B139) (**Figure 1F**) as patch-hilum materials for the genotype–phenotype study. For ease of comparison, we selected materials with clearly distinguishable seeds—those with umbilical patches and those without—for a genotype–phenotype consistency study, excluding the four materials with black testa and undetermined patches in the BC population (**Figure 1I**). Among the 71 watermelon accessions with complex genotypic germplasm resources, 22 exhibited an uncertain phenotype (**Table 2**) due to black testa and black hilum. In **Supplementary File ST1-B2**, we marked these as “without patches at hilum” according to the description of Ma (2005). Seeds with black testa also exhibited darker coloration in the hilum region compared to other parts during seed development. However, no phenotypic inconsistency was found in materials with the same genotype as B132 (female parent), which had no patches at the hilum (**Table 3**; **Supplementary File ST1**). We infer that watermelon seeds with black testa and black hilum can be considered seeds with patches at the hilum.

Watermelon seed coat color (Paudel et al., 2019; Li et al., 2020; Maragal et al., 2022) and black spots on the watermelon seed hilum are important phenotypes (Poole et al., 1941), similar to seed coat color in winter squash (*Cucurbita maxima*) (Balkaya et al., 2009), pumpkin (*Cucurbita maxima*) (Shi et al., 2021), and luffa (*Luffa* spp.) (Zhou, 2013). There are a few references concerning patches at the hilum on the testa of watermelon seeds, although research exists for other crops. Seed coat color can serve as a convenient and reliable biomarker for phenotype-based breeding/selection of cultivars (Chukwumah et al., 2009). Hilum color and testa color have been considered major factors for pea phenotype (Konstantopoulos et al., 2023). In soybean, seed hilum color and seed coat color are valuable traits for marker-assisted breeding (Yang et al., 2023; Kaňovská et al., 2024), similar to watermelon. Soybean hilum color and stem pubescence color can directly or indirectly influence plant growth, development, and yield (Zhou et al., 2024). In this study, we focused on genetic mapping for gene discovery and functional identification related to patch formation at the watermelon seed hilum, as well as genotype–phenotype analysis of the patch-hilum trait. Endogenous histological observation and developmental biology studies can help reveal the mechanisms underlying the formation of the dark-hilum spotted trait in watermelon seeds. Further research will explore the process and mechanism of patch formation at the hilum in black testa watermelon seeds.

### 3.7 Discussion of *Cla019481* gene function

*Cla019481* is annotated as a PPO gene. PPO is a copper-containing enzyme that is nearly ubiquitous in plants, animals, and fungi (Li and Steffens, 2002; Schmitz et al., 2008). It mediates multiple physiological processes, including enzymatic browning and defense against pests and pathogens (Li and Steffens, 2002). Specifically, PPO catalyzes the oxidation of polyphenols to quinones, resulting in pigment deposition and tissue discoloration (Chang, 2009; Ali et al., 2015). Although PPO is often associated with browning (Kampatsikas et al., 2017; Taranto et al., 2017), it is involved in petal color formation (Nakayamaa et al., 2001; Wang et al., 2024). PPO catalyzes the conversion of catechins to theaflavins during black tea production, forming the characteristic orange color (Balentine et al., 1997). The expression level of *MC03g0810* is positively correlated with PPO activity in the black seed coat color of bitter gourd (*Momordica* spp.) (Zhong et al., 2022). In wheat, *Ppo1* is a major gene controlling PPO activity (Zhai et al., 2023). PPO is a key enzyme contributing to the time-dependent discoloration of wheat products, and the *Ppo-D1* gene exerts the second-highest effect on grain PPO activity after the *Ppo-A1* gene (Nakamaru et al., 2025). Zhang (2021) identified the molecular mechanism underlying peel red color and flesh browning in apples with *Ma1* overexpression, showing that flesh browning at harvest is primarily caused by increased PPO activity. Collectively, these studies demonstrate that PPO and its enzymatic activity are responsible for browning in various crops/fruits.

The gene *Cla019481*, a PPO ortholog, drives melanin-like pigment deposition in the hilum region of the watermelon testa. We infer that *Cla019481* plays an important role in patch formation at the hilum of watermelon seeds, acting as the candidate gene with melanin as the principal compound responsible for the black seed coat (Li et al., 2020). Research of Roy et al. (2025) demonstrated that color deposition in common bean (*Phaseolus vulgaris* L.) originates from the hilum and spreads throughout the seed coat as the seed matures until it becomes entirely black. By analogy, we predict that black seed coat pigmentation in watermelon may similarly originate from the hilum. However, the specific role of PPO in forming patches at the umbilical part of watermelon seeds, and the mechanism by which these patches darken and contribute to the black testa during seed maturation, require further stud.

## 4 Conclusion

F_1_ generation seeds (with patches at the hilum) were derived from a cross between the female parent (without patches at the hilum) and the male parent (with patches at the hilum). The BC population was obtained by crossing F_1_ with the female parent. The segregation ratio of the patch and no-patch traits at the hilum in the BC population conformed to the expected 1:1 Mendelian ratio. A major QTL governing the stably inherited patch-hilum trait was identified on chromosome 3, spanning 5,375,019–5,784,364 bp and containing 35 annotated genes (*Cla019451* to *Cla019485*). Using a high-density, fine genetic map, a 40-kb physical genomic region was identified, harboring the candidate gene *Cla019481* for the patch-hilum trait. A reliable dCAPS marker developed within its interval demonstrated perfect genotype–phenotype co-segregation in watermelon accessions. Functional analyses confirmed that elevated genotype–phenotype expression of *Cla019481* correlates with progressive darkening of the hilum during seed development, establishing its causal role in patch formation at the hilum. Overall, *Cla019481* plays a significant role in the formation of patches at the hilum on the testa of watermelon seeds.

## Data Availability

The datasets presented in this study can be found in online repositories. The names of the repository/repositories and accession number(s) can be found in the article/**Supplementary Material**.
