# Supplementary figures and images for: Fine mapping and identification of the gene Cla019481 responsible for patches at the hilum on the testa of watermelon seeds
Source: Front Plant Sci. 2025 Dec 9;16:1680623. doi: 10.3389/fpls.2025.1680623 (PMC12722523; doi:10.3389/fpls.2025.1680623)

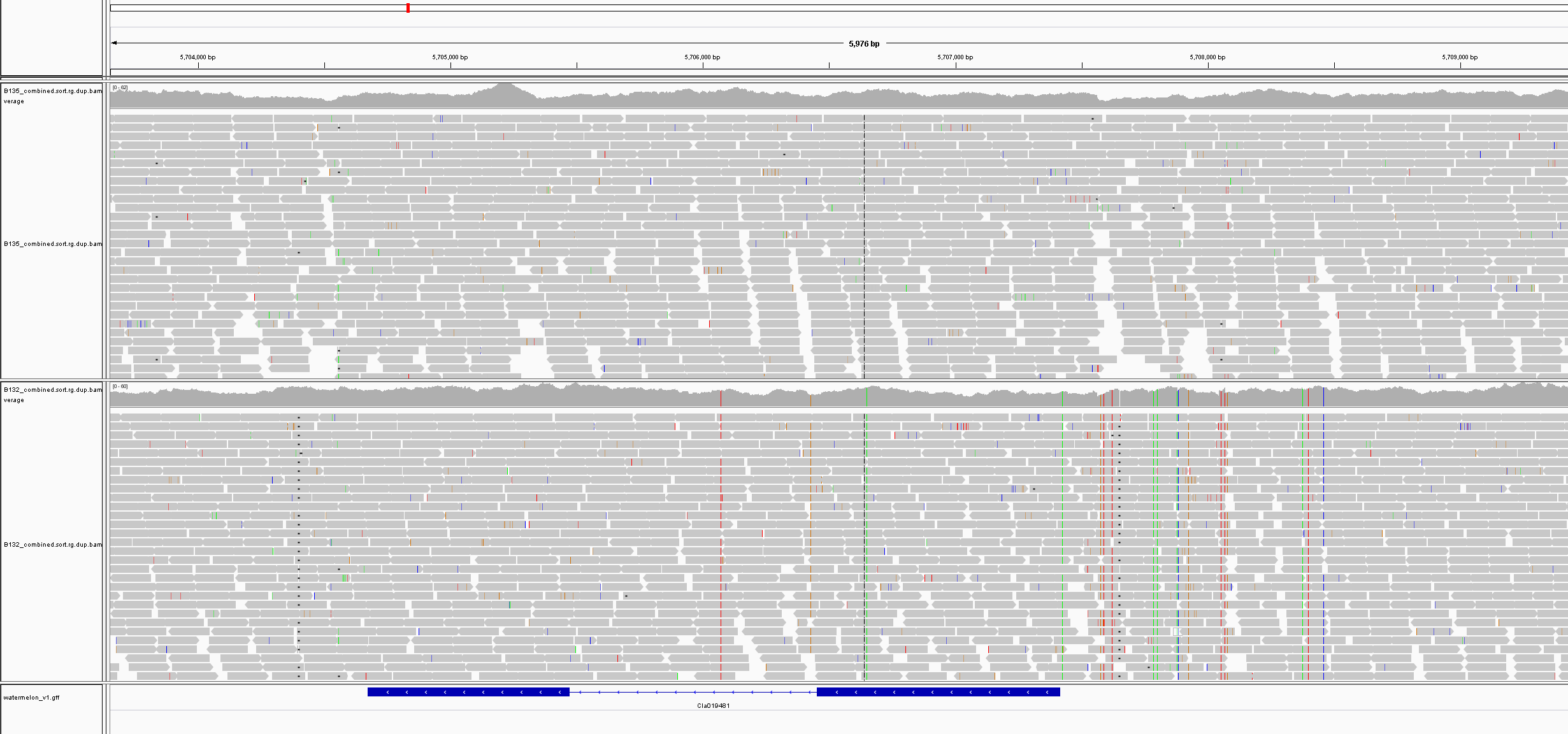

Supplement: Supplementary file 1 [file Image1.png]
