# Supplementary material for: Fine mapping and identification of the gene Cla019481 responsible for patches at the hilum on the testa of watermelon seeds
Source: Front Plant Sci. 2025 Dec 9;16:1680623. doi: 10.3389/fpls.2025.1680623 (PMC12722523; doi:10.3389/fpls.2025.1680623)
Supplement: Supplementary file 2 [file Table2.doc]

Supplementary File ST3

Primer sequences for qRT-PCR of Cla019481 and Actin

| Primer name | F_primer (5′ to 3′) | R_primer (5′ to 3′) |
| --- | --- | --- |
| Cla019481 | CGTTAGGAAAGCGGCACAATCA | TGGGAAGAACAGCCACGAGAAA |
| Actin | ATTCTCCGTTTGGACCTTGCT | TCGTAGTTTTTCTCAATGGAGG |
